# Barriers and limitations of conventional oculovisual screening methods in children: a systematic review perspective

**DOI:** 10.1186/s12886-025-04592-w

**Published:** 2026-01-19

**Authors:** Baqiatu’l Sabiqi ’Assfi Rahmat, Md Mustafa Md-Muziman-Syah, Noraishah Mohamed Nor, Thashini Sanmugam

**Affiliations:** 1 Department of Optometry, Rehabilitation and Well-being, Faculty of Health and Life Sciences, Management and Science University, Shah Alam, Selangor Malaysia; 2 Department of Optometry and Visual Science, Kulliyyah of Allied Health Sciences, International Islamic University Malaysia, Kuantan, Pahang Malaysia; 3 Children Health and Wellbeing Research Group, Kulliyyah of Allied Health Sciences, International Islamic University Malaysia, Kuantan, Pahang Malaysia; 4 Department of Nutrition Sciences, Kulliyyah of Allied Health Sciences, International Islamic University Malaysia, Kuantan, Pahang Malaysia; 5 School of Graduate Studies, Management and Science University, Shah Alam, Selangor Malaysia

**Keywords:** Barriers and limitations, Conventional vision screening, Paediatric, Systematic review

## Abstract

**Background:**

Early detection of vision problems in children is essential for preventing developmental delays and academic challenges. Conventional vision screening methods are widely implemented; however, various barriers limit their effectiveness and accessibility.

**Objective:**

This systematic review aims to identify and analyse the barriers and limitations associated with conventional vision screening methods in children.

**Methods:**

A systematic search was conducted across the Scopus, Web of Science, and PubMed databases using predefined keywords and Boolean operators. Eligible studies included empirical investigations that examined barriers and limitations in paediatric vision screening. The review adhered to PRISMA 2020 guidelines, and quality assessment was performed using the Mixed Methods Appraisal Tool (MMAT).

**Results:**

Of the studies screened, 25 met the inclusion criteria. Employing thematic analysis, five key barriers and limitations were discovered: (i) methodological limitations, (ii) resource constraints, (iii) competency gaps, (iv) socioeconomic and psychological barriers, and (v) policy and systemic challenges.

**Conclusion:**

Future research should focus on evaluating novel screening approaches that can overcome current limitations and enhance early detection rates for a broader range of paediatric vision conditions.

**Supplementary Information:**

The online version contains supplementary material available at 10.1186/s12886-025-04592-w.

## Introduction

Visual impairment in children is a significant global health issue, affecting millions and impacting their quality of life and educational outcomes. The VISION 2020 programme reported approximately 19 million children at the age of 15 and below were visually impaired, with 1.4 million classified as blind and 17.5 million experiencing low vision. The primary causes of visual impairment among children vary across regions, with the most common cause globally, accounting for over half of the cases, being refractive errors such as myopia, hyperopia, and astigmatism [1].

Myopia is the most prevalent refractive condition among children globally. A recent study reported a global myopia prevalence of 35.8% in 2023, with projections of 36.6% by 2040 and 39.8% by 2050 [2]. Early detection and appropriate intervention are crucial in preventing the development of amblyopia, reducing the risk of blindness, and controlling myopia progression in school-aged children. Untreated visual conditions can significantly affect a child’s development and academic performance [3]. Moreover, high myopia increases the risk of ocular disease that may lead to irreversible blindness, such as glaucoma, retinal detachment, and macular degeneration [4].

In response to this urgency, eye care professionals have proactively implemented vision screening programmes targeting this vulnerable age group. Vision screening refers to brief, standardised tests designed to detect oculovisual abnormalities in children before conducting a full diagnostic eye examination, and it is recommended at least once between the ages of 3 and 5 years [5]. The most commonly used screening methods include clinical test-based assessments, such as visual acuity testing and cover tests, as well as device-based screening with photoscreeners in younger children. However, this approach is resource-dependent, requiring significant manpower and time, especially for large-scale populations. In addition, these methods rely primarily on clinical signs observed in children, often overlooking the importance of early symptom reporting by parents and caregivers [6].

Previous studies reported that 6% to 28% of children have vergence anomalies, and up to 36% are diagnosed with accommodation anomalies [7, 8]. These anomalies correlate strongly with increased screen time, a trend amplified by the digital era and worsened by prolonged lockdowns during the COVID-19 pandemic [9]. Despite the prevalence of these conditions, conventional vision screening programmes focus predominantly on detecting reduced visual acuity and refractive errors, often overlooking other common vision problems in children [8, 10].

The conventional vision screening approaches provide a limited scope and may fail to detect other prevalent vision problems. These limitations emphasise the need to comprehensively evaluate existing methods to identify gaps and improve vision screening practices. Therefore, this review aims to systematically review the barriers and limitations of conventional vision screening methods in children. The review question formulated in this study applied the mnemonic of PICo, which signifies ‘P’ for Problem or Population, ‘I’ for Interest, and ‘Co’ for Context. Based on these concepts, the authors included three main aspects as part of the review: “What are the barriers and limitations (Interest) of conventional vision screening methods (Context) in children (Population)?”

## Methods

This review was systematically conducted following the Preferred Reporting Items for Systematic Review and Meta-Analysis (PRISMA) 2020 statement [11]. The protocol was registered at PROSPERO (registration number: CRD42024625325) [12].

### Eligibility criteria for studies

This review included qualitative studies that provided in-depth insight into the barriers and limitations of conventional vision screening in the paediatric population. Additionally, observational studies such as cross-sectional, cohort, or case-control studies that descriptively reported and discussed these barriers and limitations were also considered. In this context, conventional vision screening refers to the use of brief, standardised clinical test-based procedures designed to identify common paediatric oculovisual problems, including reduced visual acuity, refractive errors, strabismus, ocular abnormalities, and risk factors for developing amblyopia, prior to conducting a full diagnostic eye examination. Studies were included if the participants focused on children under 18 years, with or without underlying diseases, and reported vision screening conducted by trained personnel, who had received formal or programme-specific training in paediatric screening procedures, such as administering age-appropriate visual acuity tests, operating photoscreeners, and performing basic ocular assessments. Any studies published in languages other than English, review articles, case reports or series, editorial letters, or non-peer-reviewed opinions were excluded.

### Systematic search strategies

**Fig. 1:**
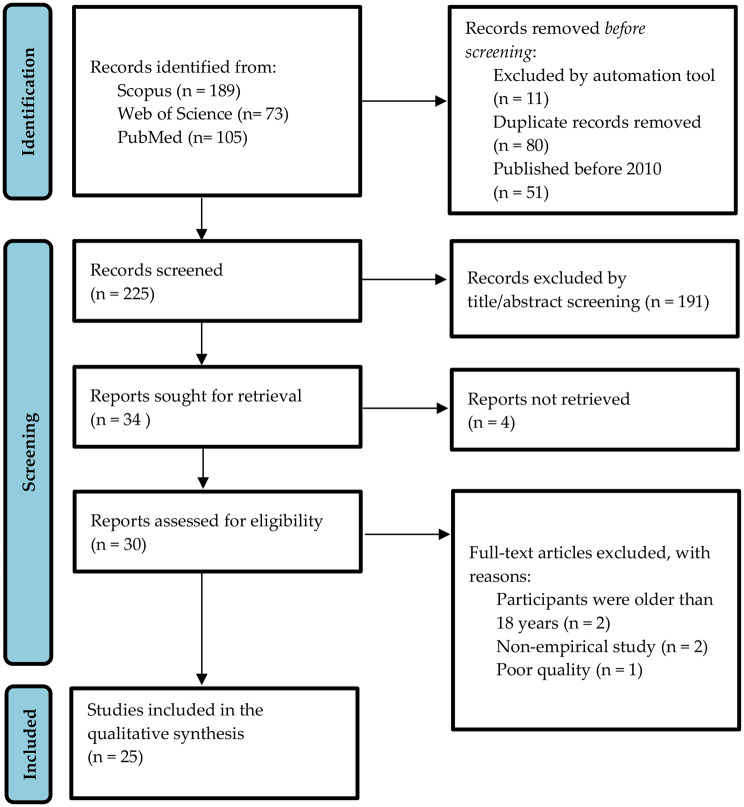
PRISMA flow diagram of study selection process

The systematic searching procedures included three processes: (i) identification, (ii) screening, and (iii) eligibility, as outlined in the PRISMA flow diagram (Fig. 1).

The identification phase involved enriching the keywords used in the search procedure. The search was conducted using the Scopus, Web of Science, and PubMed databases, with restrictions applied to English-language publications from 2010 to 2024. The search terms and strategy were adapted from a previously published, peer-reviewed systematic review protocol that adhered to the PRISMA-P (2015) guidelines [12]. In addition, the review team developed the search terms based on prior experience conducting systematic reviews that adhered to PRISMA 2020 guidelines. A combination of primary keywords, *“barriers”, “conventional”, “vision screening”,* and *“children”*, along with related terms such as *“challenge”, “difficulty”, “limitation”, “restriction”, “constraint”, “standard”, “traditional”, “routine”, “established”, “vision assessment”, “vision examination”, “eye screening”, “eye examination”, “paediatric”, “adolescent”, “schoolchildren”,* and *“preschool children”* was employed. To enhance search accuracy, Boolean operators (“OR” and “AND”), phrase searching, wildcards, truncation, and field code functions were applied across all three databases. For the PubMed search, Medical Subject Headings (MeSH) were also incorporated where applicable, such as “vision screening”[MeSH], “child”[MeSH], and combined with other keywords to enhance search sensitivity. The complete search strategies for each database are presented in Table 1.

The initial screening of studies obtained through the search process was carried out to identify and eliminate duplicate records using Mendeley reference management software. After removing duplicates, the remaining records were subjected to a title and abstract screening based on established inclusion and exclusion criteria. Studies considered potentially relevant were retrieved in full-text format for further evaluation. All full-text copies of the selected studies were stored in a designated cloud-based folder within Mendeley to enable shared access among the review authors.

Following this, two review authors (BSAR and MMMMS) independently evaluated the full texts to determine eligibility according to the predefined criteria. Any disagreements between the two reviewers were addressed through discussion, and if consensus could not be reached, a third review author (NMN) was consulted. Following the elimination of 195 articles that did not satisfy the criteria, 30 articles were retained for the subsequent selection phase. The study selection process was documented in detail to complete the PRISMA flow diagram (Fig. 1). Reasons for study exclusion at the full-text screening stage were systematically recorded in Table 2 to enhance transparency in the selection process.

**Table 1:** Search string used in selected database

Database	String
Scopus	TITLE-ABS-KEY((“barrier*” OR “challenge*” OR “difficult*” OR “limitation*” OR “restriction*” OR “constraint*”) AND (“conventional” OR “standard” OR “traditional” OR “routine” OR “establish*”) AND (“vision* screening” OR “vision* assessment*” OR “vision* examination*” OR “eye screening” OR “eye examination”) AND (“child*” OR “paediatric*” OR “kid*” OR “adolescent*” OR “schoolchildren” OR “preschool children”))
Web of Science	TS=((“barrier*” OR “challenge*” OR “difficult*” OR “limitation*” OR “restriction*” OR “constraint*”) AND (“conventional” OR “standard” OR “traditional” OR “routine” OR “establish*”) AND (“vision* screening” OR “vision* assessment*” OR “vision* examination*” OR “eye screening” OR “eye examination”) AND (“child*” OR “paediatric*” OR “kid*” OR “adolescent*” OR “schoolchildren” OR “preschool children”))
PubMed	((“vision screening“[Mesh]) AND (“child“[Mesh]) OR “paediatric“[Mesh])) AND (“barrier” OR “limitation”)

**Table 2:** Reason for exclusion of the studies

First Author, Year	Reasons
Allen, 2022	The article was a study protocol report.
Owusu-Afriyie, 2024	The study included participants aged 13 to 80 years, making it ineligible due to a lack of specific focus on the paediatric population.
Miller, 2012 Allen, 2024	The study was non-empirical. The study included participants aged over 18 years.

### Quality assessment

The methodological quality of the included studies was evaluated using the 2018 version of the Mixed Methods Appraisal Tool (MMAT), a validated instrument for appraising studies with diverse methodological approaches. The MMAT includes five distinct sets of criteria tailored to qualitative research, quantitative randomised controlled trials, quantitative non-randomised studies, quantitative descriptive studies, and mixed methods designs. Each set comprises five methodological indicators that assess core elements such as study design, data collection, analytical procedures, and interpretation. The appraisal was conducted using a structured checklist, where each criterion was rated as ‘Yes’, ‘No’, or ‘Can’t tell’. Where relevant, explanatory comments supported the assigned ratings [13].

To ensure objectivity and reduce potential bias, two independent review authors (BSAR and NMN) conducted the methodological quality assessment. The process began with an initial screening based on two core criteria: (i) clarity of the research questions and (ii) adequacy of the data in addressing those questions. Studies that did not meet these preliminary requirements were excluded from further appraisal. For studies that satisfied the initial criteria, the reviewers proceeded to evaluate each methodological domain according to the study’s specific design (see Supplementary Table).

Studies were considered eligible for inclusion in the qualitative synthesis if they attained a minimum of three affirmative ratings (≥ 3 out of 5) by consensus among the two primary review authors (BSAR and NMN). In cases of disagreement regarding study inclusion, a third review author (MMMMS) served as an arbiter to resolve discrepancies. Inter-rater reliability was assessed using Cohen’s kappa (κ = 0.649), reflecting a good level of agreement between reviewers. This rigorous evaluation process ensured that only high-quality studies were incorporated into the qualitative synthesis, thus enhancing the credibility and validity of the review’s findings. Following this assessment, all authors reached a consensus that 25 studies met the minimum methodological quality (see Table 3).

**Table 3:** Results of the quality assessment

First author, Year	Study design	*QA1	*QA2	*QA3	*QA4	*QA5	No. of criteria	Inclusion
Collins, 2022	MX	Y	N	Y	N	N	2/5	N
Vilà-de Muga, 2021	QN-NR	Y	Y	C	C	Y	3/5	Y
Raoof, 2016	QN-DC	Y	C	Y	N	Y	3/5	Y
Wilhelmsen, 2022	QN-DC	Y	C	Y	C	Y	3/5	Y
Ademola-Popoola, 2021	QN-DC	Y	Y	Y	C	Y	4/5	Y
Furr, 2018	QN-NR	Y	Y	Y	N	Y	4/5	Y
Barugel, 2019	QN-DC	Y	C	Y	C	Y	3/5	Y
Sharma, 2020	QN-DC	Y	N	Y	C	Y	3/5	Y
Sun, 2016	QN-DC	Y	Y	Y	C	Y	4/5	Y
Alrasheed, 2016	MX	Y	Y	Y	N	Y	4/5	Y
Ravid-Saffir, 2023	QL	Y	Y	Y	Y	Y	5/5	Y
Yashadhana, 2021	QL	Y	Y	Y	Y	Y	5/5	Y
Lohfeld, 2021	QL	Y	Y	Y	Y	Y	5/5	Y
Teerawattananon, 2014	MX	Y	Y	Y	Y	Y	5/5	Y
Prakash, 2024	MX	Y	Y	Y	Y	Y	5/5	Y
Jindani, 2021	QN-DC	Y	C	Y	C	Y	3/5	Y
Le, 2018	QN-DC	Y	C	Y	N	Y	3/5	Y
Marsh-Tootle, 2012	QL	Y	Y	Y	Y	Y	5/5	Y
Marsh-Tootle, 2010	QN-DC	Y	C	Y	C	Y	3/5	Y
Vongsachang, 2020	QL	Y	Y	Y	Y	Y	5/5	Y
Alsaqr, 2023	QN-DC	Y	C	Y	N	Y	3/5	Y
Su, 2013	QN-DC	Y	Y	Y	N	C	3/5	Y
Noma, 2011	QN-DC	Y	C	Y	C	Y	3/5	Y
Donaldson, 2018	QN-DC	Y	C	Y	C	Y	3/5	Y
Ebeigbe, 2018	QL	Y	Y	Y	Y	Y	5/5	Y
Masarwa, 2023	QN-DC	Y	N	Y	C	Y	3/5	Y

QA = Quality assessment; QN-DC = Quantitative descriptive; QN-NR = Quantitative non-randomised; QL = Qualitative; MX = Mixed-method; Y = Yes; N = No; C = Can’t tell

*Full questions described in the Mixed Method Appraisal Tool (MMAT) (see Supplementary Table)

### Data extraction and analysis

A review author (BSAR) extracted data from each included study into Microsoft Word using a structured table format, and the extracted data was independently verified by another review author (MMMMS). The data extraction process systematically documented the characteristics of the included studies, encompassing the following areas: (i) General information, including the first author, publication year, funding sources, and authors’ conflicts of interest; (ii) Study setting, detailing study design, location, country, vision screening method, and the screener; (iii) Population, specifying the age range and number of participants; (iv) Study aims; and (v) Outcomes, where findings relevant to the identified barriers and limitations were aligned with the review question.

The extracted data primarily focused on identifying recurring barriers and limitations related to conventional vision screening methods in paediatric populations. Any discrepancies during the extraction process were resolved through discussion between the reviewers, and when consensus could not be reached, a third review author (NMN) was consulted. This systematic documentation process facilitated a comprehensive synthesis of the evidence and ensured a structured and transparent approach to data management for the qualitative analysis.

The data extracted from the included studies were analysed using thematic analysis. The reviewers first familiarised themselves with the dataset through repeated readings of the extracted findings. Inductive open coding was then carried out to identify meaningful features within the data, and these initial codes were subsequently examined and grouped into broader categories through an axial coding process. Emerging themes were reviewed and refined collaboratively to ensure internal consistency, coherence, and an accurate representation of the full dataset, with any disagreements resolved through discussion. Each theme was then clearly defined and named, and an analytic narrative was developed to explain its relevance to the review’s objectives. Finally, the findings were synthesised into a structured report [12, 14].

## Results

The database searches identified 367 records, including 189 from Scopus, 73 from Web of Science, and 105 from PubMed. Four reports could not be retrieved after eliminating 80 duplicates and excluding 253 based on title/abstract screenings; 30 full-text articles were evaluated for eligibility criteria and quality assessment. Of these, four were excluded based on eligibility criteria and one due to quality assessment, resulting in the inclusion of 25 studies for the qualitative synthesis.

### Characteristics of the included studies

A total of 25 studies were included, covering various vision screening methods and target populations (Table 4). Among them, seven studies (7/25) were conducted in Asia [15–21], three studies (3/25) were conducted in Europe [22–24], seven studies (7/25) were conducted in North America [25–31], two studies (2/25) were conducted in South America [32, 33], five studies (5/25) were conducted in Africa [34–38] and one study (1/25) were conducted in Oceania [39].

The included studies were categorised into clinical-based, school-based, and community-based settings (Table 4). Clinical-based studies involved screening conducted in hospitals, eye clinics, or primary care facilities and utilised both device-based and clinical test–based approaches. Device-based methods primarily relied on photoscreeners, particularly the SPOT Vision Screener, to detect refractive errors and risk factors for amblyopia in young children [15, 22, 23, 35]. Clinical test–based approaches in these settings included red reflex screening [17, 39], visual acuity testing [26, 27]), and additional assessments such as external eye inspection [28], refraction testing [25], and measurements of stereopsis [29]. These assessments were typically administered by health care professionals, including primary care providers and eye care practitioners.

School-based studies focused on screening programmes implemented within preschool and school environments and largely examined barriers, facilitators, and programme implementation rather than test accuracy. These studies targeted early childhood and school-aged populations across various regions [16, 19, 21, 30–34, 36–38]. Some school-based programmes incorporated visual acuity testing and binocular vision assessments conducted by trained teachers [16, 34]. In contrast, community-based studies were limited to those conducted outside formal school or clinical settings and did not report the implementation of structured screening protocols. These studies primarily explored parental perspectives, health beliefs, and barriers to accessing eye care or vision screening services [18, 20, 24].

Moreover, most of the included studies targeted different age groups, ranging from newborns to school-aged children. Studies focusing on infants (newborns to six months) were conducted in China [17, 39] and New Zealand [39]. Preschool vision screening for children aged 3 to 6 years was conducted in the United States [26, 27], Spain [22], and India [15]. School-based vision screening targeted children aged 5 to 12 years in Nigeria [37], Saudi Arabia [20], and Canada [31]. Only one study specifically focused on children with special educational needs, which was performed in India [21].

### The emerging themes

The thematic analysis synthesised the findings of barriers and limitations reported across the included studies into five main themes: (i) methodological limitations, (ii) resource constraints, (iii) competency gaps, (iv) socioeconomic and psychological barriers, and (v) policy and systemic challenges (Table 5).

#### Methodological limitations

The first theme highlights the methodological limitations associated with vision screening practices. One prominent issue is age-related variability in test sensitivity. Photoscreening tools have been shown to demonstrate lower sensitivity in younger children. Specifically, one study (1/25, 4%) reported approximately 25% lower sensitivity in children aged 18 to 23 months compared with those aged 24 to 30 months [22].

In addition, several studies have pointed to the limited detection capabilities of certain vision abnormalities. The SPOT™ vision screener has been reported to be ineffective in detecting strabismus and hypermetropia, with sensitivities as low as 42% and 36%, respectively [15, 22].

Measuring visual acuity remains a challenge, particularly in children under 4 years. Two studies (2/25, 8%) reported reduced test reliability or the feasibility of conventional methods, such as the LEA Symbols test in younger children due to poor cooperation and comprehension [22, 29] In addition, two studies (2/25, 8%) identified screening environment-related factors, including the unsuitability of tabletop devices [29] and inadequate classroom lighting, as factors that compromised screening accuracy [34].

#### Resource constraints

Resource constraints pose significant challenges to the effective implementation of paediatric vision screening programmes. A recurring issue is the limited capacity within health systems. The Tanzanian health system struggles to provide comprehensive screening programmes that incorporate multiple recommended screening components rather than a single test for all children. This has necessitated adopting alternative strategies, including teacher-led screenings [34]. Similarly, the insufficient coverage of vision screening programmes by health care practitioners results in increased reliance on teachers to carry out screenings [16, 40]. Additionally, resource limitations persist, including inadequate access to essential screening equipment [39].

Inadequate reimbursement further exacerbates these challenges. Health care practitioners frequently find it difficult to justify the time and effort required for comprehensive vision screenings when financial reimbursement does not align with their workloads [26, 28]. Financial barriers also affect families, as limited insurance coverage for routine vision screenings places an added burden on parents seeking follow-up care [26]. Furthermore, time constraints hinder primary care practitioners, including paediatricians, general practitioners, and primary care nurses, from conducting comprehensive vision screening. Limited chair time during routine well-child clinic visits was identified as a significant barrier to the provision of these services [26, 39].

#### Competency gaps

Insufficient training and gaps in knowledge among vision screeners represent a major barrier to the effective delivery of paediatric vision screening programmes. Approximately 20% of surveyed general medical practitioners and nurses lacked confidence in interpreting red reflex screening results [39]. This is an indication of broader deficiencies in clinical knowledge among personnel conducting vision screenings.

A key factor contributing to this issue is the lack of formal training. The same study revealed that only 17.3% of respondents had received adequate instruction in performing red reflex screening [39]. Similarly, limited training among general medical practitioners in executing essential screening procedures, including the corneal light reflex and cover-uncover tests [28], This gap extends to non-health care vision screeners, namely schoolteachers and community health workers, who also frequently lack structured training, as highlighted by Ademola-Popoola and colleagues in 2021 [35].

#### Socioeconomic and psychological barriers

Socioeconomic disparities and cultural perceptions play a significant role in shaping access to and participation in paediatric vision screening programmes. Financial barriers are among the most prominent barriers. Certain studies have shown that high costs associated with clinic-based vision assessments and follow-up eye examinations conducted by eye care professionals often discourage families from seeking vision screening and completing subsequent care [18, 41]. This issue is further compounded by inadequate health insurance coverage, with a substantial proportion of students lacking insurance for eye care services [37].

Parental beliefs also exert a critical influence on screening uptake. Common misconceptions, including underestimating the need for vision screening, fear of children wearing glasses, and mistrust of eye care professionals, frequently act as deterrents [18, 38]. Additionally, some parents fail to perceive their child’s vision issues as serious, particularly when symptoms are not overt, which contributes to poor follow-up rates [36]. Cultural stigma associated with spectacle use and distrust in health services, further exacerbates the reluctance to engage with screening services [33].

A general lack of awareness about the importance of early vision screening remains a major hindrance to timely detection and intervention. Multiple studies have emphasised that many parents, caregivers, and even health care practitioners are unaware of the critical role vision screening plays in early childhood development [18–20, 24, 27, 30, 31, 34, 37, 38]. There is a common belief that vision problems will resolve naturally or be identified during routine checkups. Additionally, some health care practitioners deprioritise vision screening in paediatric care, considering it less urgent than other medical assessments [39].

#### Policy and systemic challenges

Policy and systemic issues represent fundamental barriers to the standardisation and effective implementation of paediatric vision screening programmes. Regional disparities in programme delivery result in unequal access to eye care services for children. For example, one study reported notable inconsistencies in Canada, where some public health units fail to implement mandated universal screening programmes despite the existence of clear national guidelines [31]. These inconsistencies contribute to fragmented service delivery and limit the overall impact of screening initiatives.

Inconsistent adherence to recommended screening protocols is another major concern. The discrepancies between self-reported and actual screening practices among health care providers indicate a lack of uniformity in programme execution. Despite efforts to improve standardisation, a universally accepted, evidence-based framework remains lacking [25]. The development and adoption of a structured framework has been strongly recommended to promote equitable access and consistent quality of care [31].

Effective implementation also depends on intersectoral collaboration, particularly between health and education ministries. However, this coordination is often insufficient. Integrated delivery models in school-based vision care are essential, as a lack of cross-sector collaboration hinders programme scalability and long-term effectiveness [33].

Legal and regulatory barriers further complicate the development of comprehensive screening systems. Unclear or restrictive regulations can hinder the expansion of vision screening services, particularly in non-clinical settings [23].

**Table 4:** Characteristics of included studies

First author, Year	Place, Country	Vision screening method	Intended age group	Participants of the study	Aim of the study
**Clinical-based**					
Sharma, 2020	NR, India	Photoscreening (Spot™ vision screener)	6 months – 5 years	219 children	To evaluate the accuracy of Spot™ vision screener as a noncycloplegic photorefractor in detecting amblyopia risk factors in preschool children.
Vilà-de Muga, 2021	Barcelona, Spain	Photoscreening (Spot™ vision screener)	18–30 months	453 children	To assess the usefulness of the photoscreening for young children.
Barugel, 2019	NR, France	Photoscreening (Spot™ vision screener)	4 years above	41 children aged 4 years above	To evaluate the effectiveness of the Spot™ vision screener in detecting refractive errors among children with limited access to ocular health care.
Marsh-Tootle, 2012	NR, United States	VA test (Vision in Preschooler Screening Kit & Eye Check Screening Test)	3–6 years	13 physicians and 32 nurses or certified medical assistants	To investigate the practices, barriers, and facilitators of universal preschool vision screening (PVS) at paediatric primary care offices.
Su, 2013	NR, United States	VA test	3–14 years	58 parents	To identify barriers to follow-up eye care in children who failed a VA screening conducted by their primary care provider.
Le, 2018	Ontario, Canada	Visual inspection of external eye structures, red reflex, fix and follow, pupillary reaction to light, corneal light reflex, cover test, and VA tests	Children	Primary care physicians	To assess how well primary care physicians in Ontario follow the vision screening guidelines for children as recommended by the Canadian Paediatric Society and the Rourke Baby Record. It also seeks to identify barriers to vision screening in the primary care setting.
Sun, 2016	NR, China	Red-reflex screening	Newborns (1 week)	Newborns	To validate the sensitivity of an isolated red reflex test in detecting ocular abnormalities of the anterior and posterior segments in newborns.
Raoof, 2016	NR, New Zealand	Red reflex screening	Newborns – 6 weeks (infant)	484 respondents (267 GPs, 153 midwives, and 50 paediatricians)	To survey the current state of red reflex screening practices and attitudes among health care practitioners.
Ademola-Popoola, 2021	Kwara, Nigeria	Interview-based, Bruckner red reflex, photoscreening (Spot™ vision screener	Newborn – 2 years	Trained Community health workers screened 5,609 children	To evaluate the effectiveness and limitations of different vision screening methods (interview, Bruckner test, and instrument screener) in the early detection
Furr, 2018	Michigan, United States	Titmus V3 vision screener, VA (LEA Symbols test), and stereopsis tests (Stereofly)	36–66 months	263 preschool children	To evaluate the testability of the Titmus V3 vision screener as an alternative method for screening VA and strabismus in preschool children.
Marsh-Tootle, 2010	NR, United States	VA, stereopsis, and refraction (autorefractors) tests	3–4 years	53 participants consist of paediatricians, physicians, and nurses	To evaluate the knowledge, attitudes, and environment of primary care providers and to develop a conceptual framework showing their impact on self-reported preschool vision screening behaviours.
**School-based**					
Ravid-Saffir, 2023	NR, Israel	NR	Preschool children	Panel of experts consists of paediatric ophthalmologists, optometrists, orthoptists, paediatricians, and social science officers	To develop and validate a questionnaire for assessing parents’ awareness, perception, and health literacy regarding children’s vision tests.
Yashadhana, 2021	Bogotá, Colombia	NR	Schoolchildren	37 participants consist of government stakeholder, NGOs, ECPs, teachers, school nurses, parents, and children	To identify barriers and enablers to accessing school-based eye health programmes in Bogotá, Colombia.
Lohfeld, 2021	Cross River, Nigeria	NR	Schoolchildren	Parents	To understand the reasons for parents’ non-adherence to referrals for follow-up eye examinations for their children in Cross River State, Nigeria.
Vongsachang, 2020	NR, United States	NR	Schoolchildren	90 parents and 117 teachers/staff	To examine factors that decrease participation in school-based vision programmes from the perspectives of parents and teachers.
Prakash, 2024	Hyderabad, India	NR	Children with SEN.	Special school managers, eye health programme organizers	To identify the awareness level among special school managers regarding the eye health needs of children with SEN and to explore the barriers to organizing School Eye Health (SEH) programmes in special schools.
Jindani, 2021	NR, Canada	NR	Early childhood	Medical officers of health and 34 public health units	To assess the existence and uptake of school-based vision screening programmes across Canada.
Ebeigbe, 2018	NR, Nigeria	NR	5–12 years	35 parents and 10 ECPs	To assist eye care professionals in planning better programme regimens and to help identify various elements that either facilitate or hinder eye care seeking behaviour of parents for their school-age children.
Alrasheed, 2016	South Darfur, Sudan	NR	Children (not specify age)	Children 12–17 years and parents	To assess the level of knowledge, attitudes, and practices (KAPs) of students and their parents regarding childhood eye services and barriers to accessing child eye care.
Teerawattananon, 2014	NR, Thailand	VA and refraction tests	Preschool and primary schoolchildren	Teachers (screeners), children, and parents	To assess the accuracy and feasibility of a refractive error screening programme conducted by teachers in preschool and primary schools in Thailand.
Wilhelmsen, 2022	NR, Tanzania	VA, AA and NPC tests	Primary schoolchildren of Standard 5 (10–12 years)	104 pupils from Standard 5	To highlight the importance of vision screening by teachers in schools to ensure that children with vision problems are identified and supported.
Noma, 2011	São Paulo, Brazil	VA test and external inspection	Schoolchildren	Schoolchildren, parents/guardians	To identify barriers to attendance for eye examinations of schoolchildren.
**Community-based**					
Alsaqr, 2023	NR, Saudi Arabia	NR	3–7 years	1037 parents of children aged 3–7 years	To evaluate parental perspectives on accessing eye care for children under 7 years old.
Donaldson, 2018	London, United Kingdom	NR	4–6 years	Parents of children 4–6 years	To investigate the attitudes of parents toward eye care for their young children and to explore possible barriers to accessing eye care for this age group.
Masarwa, 2023	Ashkelon, Israel	VA test	3–5 years	100 parents	To explore the role of parental health beliefs in seeking eye examinations for children. It sought to understand what drives parents to seek eye care for their children and how parental perceptions influence this decision-making process.

*NR = Not reported; VA = Visual acuity; AA = Amplitude of accommodation; NPC = Near point of convergence; GPs = General practitioners;

NGOs = Non-governmental organizations; ECPs = Eye care practitioners; SEN = Special educational need

**Table 5:** Themes and subthemes of barriers and limitations associated with vision screening

Themes	Subthemes	First Author, Year
Methodological Limitations	1. Variability in Sensitivity Across Different Age Groups	Vilà-de Muga, 2021, Furr, 2018
	2. Limited Detection of Certain Conditions	Vilà-de Muga, 2021, Barugel, 2019, Sharma, 2020, Sun, 2016, Ademola-Popoola, 2021
	3. Challenges in Measuring Visual Acuity and Strabismus	Vilà-de Muga, 2021, Furr, 2018, Alsaqr, 2023
	4. Testing Setting Issue	Furr, 2018, Wilhelmsen, 2022
Resource Constraints	1. Limited Capacity of Health Systems	Wilhelmsen, 2022, Teerawattananon, 2014, Prakash, 2024
	2. Lack Coverage of Vision Screening Program	Teerawattananon, 2014, Yashadhana, 2021, Marsh-Tootle, 2012
	3. Lack of Equipment	Raoof, 2016
	4. Inadequate Reimbursement	Le, 2018, Marsh-Tootle, 2012, Marsh-Tootle, 2010
	5. Time Constraints	Raoof, 2016, Le, 2018, Marsh-Tootle, 2012, Marsh-Tootle, 2010
Competency Gaps	1. Lack of Clinical Confidence	Raoof, 2016
	2. Lack of Training	Raoof, 2016, Ademola-Popoola, 2021, Le, 2018, Teerawattananon, 2014, Wilhelmsen, 2022
Socioeconomic and Psychological Barriers	1. Cost Barriers	Alrasheed, 2016, Ebeigbe, 2018, Masarwa, 2023, Donaldson, 2018
	2. Parental Perceptions and Beliefs	Alrasheed, 2016, Lohfeld, 2021, Noma, 2011, Masarwa, 2023, Donaldson, 2018
	3. Socioeconomic Disparities	Yashadhana, 2021, Ebeigbe, 2018, Masarwa, 2023, Alsaqr, 2023
	4. Lack of Awareness among Parents and Practitioners	Wilhelmsen, 2022, Alrasheed, 2016, Ravid-Saffir, 2023, Jindani, 2021, Alsaqr, 2023, Vongsachang, 2020, Su, 2013, Donaldson, 2018, Ebeigbe, 2018, Masarwa, 2023, Raoof, 2016
Policy and Systemic Challenges	1. Lack of Standardisation	Jindani, 2021, Donaldson, 2018
	2. Policy Gaps and Implementation Barriers	Jindani, 2021, Marsh-Tootle 2010, Donaldson, 2018
	3. Collaboration and Coordination Issues	Yashadhana, 2021, Noma, 2011
	4. Legal and Regulatory Constraints	Barugel, 2019

## Discussion

This systematic review synthesises evidence across five major themes: (i) methodological limitations, (ii) resource constraints, (iii) competency gaps, (iv) socioeconomic influence and parental perceptions, and (v) policy and systemic challenges, revealing the multifactorial nature of the barriers and limitations in conventional paediatric vision screening. The results of this review align with previous research that highlights the shortcomings of conventional vision screening methods for children [41–43].

The results of the current review highlight methodological limitations in conventional paediatric vision screening practices, raising critical concerns regarding the reliability, sensitivity, and overall appropriateness of the tools and procedures currently in use. The findings align with prior evidence emphasising the shortcomings of standardised screening protocols when applied across diverse paediatric age groups [10]. Particularly, the reduced sensitivity of photoscreening tools in younger children underscores a critical vulnerability in early detection efforts, especially during a period when timely identification of amblyogenic risk factors is most crucial [22].

The poor detection rates of conditions like strabismus and hypermetropia further underscore the limitations of device-based screeners [15, 22]. These findings align with concerns raised in other studies, which note that even advanced tools often fail to identify subtle or intermittent ocular deviations without comprehensive binocular function testing [44]. The implications concern the context of public health, where screening programmes often rely heavily on these tools due to their ease of use, despite suboptimal diagnostic performance in certain age groups and conditions.

Moreover, the challenge of obtaining reliable visual acuity measurements in children under 4 years old, due to limited attention span, poor cooperation, and variable cognitive development, highlights a persistent gap in screening effectiveness. While tools like the LEA symbols test are considered more child-friendly than Snellen charts, their success still depends heavily on the child’s ability to understand and engage with the task [29]. This issue is particularly pronounced in non-clinical settings, such as schools and community centres, where environmental factors such as inadequate lighting and distractions further compromise test reliability [34].

Resource constraints represent a persistent and multifaceted barrier to the successful implementation of paediatric vision screening programmes, particularly in low-income and rural settings. In many of these communities, systemic underinvestment in health care infrastructure results in a shortage of trained personnel and insufficient access to appropriate screening equipment [16, 34]. As a compensatory measure, teacher-led screenings have emerged as a common approach to broaden coverage. While this strategy may increase reach, it often comes at the expense of diagnostic accuracy. Evidence suggests that teachers, though well-positioned within the school environment, frequently lack the necessary training and clinical acumen to identify subtle or complex visual impairments, leading to both false positives and missed cases [35].

In parallel, financial disincentives further compound the issue. Inadequate reimbursement structures discourage health care providers from dedicating time and resources to vision screening during routine paediatric visits [26]. This is particularly problematic in settings where provider time is already stretched thin, and screening is deprioritised in favour of other health concerns. Consequently, children who require early detection and timely intervention are left vulnerable to delayed diagnosis and preventable vision loss.

The implications suggest that resource limitations do not merely restrict access but may also impact the quality, consistency, and credibility of screening programmes. Addressing this challenge requires systemic investment in health care delivery models that support vision screening as an essential component of child health. This includes funding for portable, low-cost screening tools, structured training programmes for both clinical and non-clinical screeners, and reimbursement policies that incentivise comprehensive vision assessments within routine care. Future research should also focus on evaluating cost-effective, scalable solutions, particularly those leveraging technology or community-based task-shifting models that have the potential to expand equitable access to high-quality vision screening services.

This review found a significant impact of competency gaps on the effectiveness and accuracy of paediatric vision screening. A consistent theme across studies is the lack of clinical confidence and formal training among providers in performing and interpreting essential vision screening tests. In a survey conducted in New Zealand involving 484 health care providers, including general practitioners and paediatricians, nearly one in five respondents reported a lack of confidence in interpreting red reflex test findings [39]. Similarly, a study reported that even common screening procedures, such as the corneal light reflex and cover-uncover tests, are underutilised or poorly performed due to insufficient training among physicians [28].

These findings reflect broader concerns raised in the literature regarding the variability of medical education in both undergraduate medical curricula and graduate continuing professional development programmes [45]. Without a consistent, evidence-based training framework, vision screening often becomes a mere formality instead of a crucial clinical task function. This situation is further exacerbated in contexts where vision screening is delegated to teachers or community health workers (CHWs). Although these stakeholders are instrumental in expanding access, particularly in low-resource settings, their lack of specialised training can result in misidentification of vision problems, delayed referrals, and diminished trust in screening outcomes [35].

Socioeconomic and psychological barriers were identified as pivotal factors limiting the accessibility, uptake, and effectiveness of paediatric vision screening programmes. These findings corroborate previous studies that emphasise the role of social determinants in shaping health care access and utilisation [46]. The evidence synthesised here demonstrates that families from lower socioeconomic backgrounds face disproportionately high structural and perceptual barriers to vision care, thereby exacerbating existing health inequities.

High treatment costs and limited insurance coverage are recurring themes. The financial burdens placed on families, which act as substantial barriers to attending initial screening attendance and follow-up care [18, 37, 38]. These challenges are particularly acute in contexts where vision screening is not publicly funded or included in standard child health packages, leaving families to navigate out-of-pocket costs for both diagnosis and corrective interventions. Such financial constraints have also been reported in other areas of child health, including dental and developmental services, further reinforcing the interconnectedness of socioeconomic status and health outcomes [47].

Psychological and cultural factors also critically influence parental decision-making. Misperceptions about the severity of vision problems, stigma associated with wearing spectacles, and general mistrust of eye care providers contribute to reduced compliance with screening programmes [36, 40]. These findings echo earlier research showing that health beliefs, cultural norms, and low health literacy can serve as invisible but formidable barriers to care-seeking behaviour [48].

Compounding these issues is the widespread lack of awareness regarding the purpose and importance of early vision screening. Across multiple studies, both caregivers and some health care providers underestimated the value of routine screening, often assuming that vision issues would self-correct or become apparent through academic or behavioural problems [19, 27, 34]. This lack of awareness not only delays diagnosis and treatment but also undermines the credibility of screening initiatives.

This review reveals that policy and systemic barriers play a fundamental role in perpetuating disparities in the delivery and accessibility of paediatric vision screening services. While some countries have established formal guidelines and mandates for vision screening, their implementation of these policies is often fragmented and inconsistent, resulting in significant regional and socioeconomic inequities.

Although universal vision screening is mandated in Canada, its implementation and enforcement vary significantly across provinces [31]. In some regions, public health units fail to deliver the mandated services due to funding constraints, workforce shortages, or lack of oversight. This regional variability highlights a common challenge faced by many health care systems, stemming from the disconnect between national policy and local implementation. Without mechanisms to ensure compliance, monitor quality, and evaluate outcomes, even well-intentioned policies can fail to achieve their goals. This challenge is not unique to Canada; it mirrors the difficulties encountered in low- and middle-income countries as they strive to implement effective screening programmes for various health conditions [49]. Barriers to health care access are not solely defined by geographical or economic constraints but also encompass a spectrum of factors, including awareness, education, and effective communication of the risks associated with vision impairment [50].

Legal and regulatory frameworks also influence the scope and quality of vision screening. The restrictions on the use of cycloplegic refraction in certain jurisdictions limit the diagnostic accuracy of screenings, especially in detecting latent hyperopia and other refractive errors [23]. These legal limitations may be rooted in professional scope-of-practice regulations or concerns about the administration of pharmaceutical agents by non-specialists. However, in practice, they hinder comprehensive eye assessments and contribute to variability in care standards across regions. The absence of a unified strategy undermines efforts to create sustainable, equitable, and population-wide screening models [40].

While the reviewed evidence provides valuable insights, several limitations must be acknowledged. First, only English-language publications were included, which may have resulted in language bias and the exclusion of relevant studies published in other languages. Second, many of the included studies were qualitative or cross-sectional, limiting the ability to infer causality. In addition, a quantitative synthesis or meta-analysis was not feasible due to substantial heterogeneity across study designs, screening settings, participant characteristics, screening tools, and reported outcomes; consequently, the findings were synthesised qualitatively, which limits the ability to estimate pooled effects or to make direct comparisons across studies.

The findings of this review have essential clinical, policy, and research implications. In clinical practice, the findings indicate a need to strengthen training opportunities for health care providers to better equip them with the knowledge and skills required for paediatric vision screening techniques. Multimodal screening approaches that combine device-based tools with clinical tests may improve detection accuracy across a broader range of vision disorders and could be considered when resources permit.

From a policy perspective, the findings highlight the potential value of developing more standardised vision screening protocols to help reduce regional variation in practice. School-based vision screening programmes may provide a useful platform, particularly in low-resource settings, as schools can serve as accessible points for early vision care. In addition, increased attention to funding mechanisms and insurance coverage for paediatric vision assessments may help alleviate financial barriers faced by families and improve access to follow-up care.

Future research should focus on addressing current gaps, particularly through longitudinal studies that track follow-up adherence and treatment outcomes. Additional investigations into parental perceptions and psychosocial barriers would provide deeper insights into the cultural factors affecting vision screening participation. Furthermore, while this review focused on barriers and limitations associated with conventional oculovisual screening, emerging evidence indicates that cerebral visual impairment (CVI) is increasingly recognised as a cause of visual dysfunction in children. Current screening approaches primarily emphasise ocular-based assessments and may not adequately identify visual processing difficulties. Future research should therefore explore the feasibility, validity, and implementation challenges of incorporating screening for visuoperceptual deficits (VPD) alongside ocular screening, particularly in populations at higher risk of neurodevelopmental impairment.

## Conclusion

The barriers and limitations identified in this review underscore the challenges faced in implementing effective paediatric vision screening programmes. Device-related inaccuracies, resource constraints, inadequate training, socioeconomic disparities, and systemic inefficiencies all contribute to gaps in early detection and intervention. Addressing these challenges requires a comprehensive and collaborative approach, including improved training for screening personnel, enhanced awareness campaigns for parents, increased investment in screening infrastructure, and policy reforms to standardise and expand vision screening programmes globally.

## Supplementary Information

Below is the link to the electronic supplementary material.

Supplementary Material 1

## Data Availability

All data generated or analysed during this study are included in this published article and its supplementary materials. Further information is available from the corresponding author upon reasonable request.
