# Supplementary material for: Barriers and limitations of conventional oculovisual screening methods in children: a systematic review perspective
Source: BMC Ophthalmol. 2026 Jan 19;26:64. doi: 10.1186/s12886-025-04592-w (PMC12874825; doi:10.1186/s12886-025-04592-w)
Supplement: Supplementary file 1 — Supplementary Material 1 [file 12886_2025_4592_MOESM1_ESM.docx]

**Supplementary Table. The criteria used to evaluate the methodology and analysis used in the selected articles**

| **Study designs** | **Methodological quality criteria** |
| --- | --- |
| Screening questions (for all types) | QA1. Are there clear research questions? |
|  | QA2. Do the collected data allow to address the research questions? |
|  | Further appraisal may not be feasible or appropriate when the answer is ‘No’ or ‘Can’t tell’ to one or both screening questions. |
| Qualitative | QA1. Is the qualitative approach appropriate to answer the research question? |
|  | QA2. Are the qualitative data collection methods adequate to address the research question? |
|  | QA3. Are the findings adequately derived from the data? |
|  | QA4. Is the interpretation of results sufficiently substantiated by data? |
|  | QA5. Is there coherence between qualitative data sources, collection, analysis and interpretation? |
| Quantitative randomized controlled trials | QA1. Is randomization appropriately performed? |
|  | QA2. Are the groups comparable at baseline? |
|  | QA3. Are there complete outcome data? |
|  | QA4. Are outcome assessors blinded to the intervention provided? |
|  | QA5 Did the participants adhere to the assigned intervention? |
| Quantitative non- randomized | QA1. Are the participants representative of the target population? |
|  | QA2. Are measurements appropriate regarding both the outcome and intervention (or exposure)? |
|  | QA3. Are there complete outcome data? |
|  | QA4. Are the confounders accounted for in the design and analysis? |
|  | QA5. During the study period, is the intervention administered (or exposure occurred) as intended? |
| Quantitative descriptive | QA1. Is the sampling strategy relevant to address the research question? |
|  | QA2. Is the sample representative of the target population? |
|  | QA3. Are the measurements appropriate? |
|  | QA4. Is the risk of nonresponse bias low? |
|  | QA5. Is the statistical analysis appropriate to answer the research question? |
| Mixed methods | QA1. Is there an adequate rationale for using a mixed methods design to address the research question? |
|  | QA2. Are the different components of the study effectively integrated to answer the research question? |
|  | QA3. Are the outputs of the integration of qualitative and quantitative components adequately interpreted? |
|  | QA4. Are divergences and inconsistencies between quantitative and qualitative results adequately addressed? |
|  | QA5. Do the different components of the study adhere to the quality criteria of each tradition of the methods involved? |

Source: Hong (2018)
